# Effect of the micronutrient iodine in thyroid carcinoma angiogenesis

**DOI:** 10.18632/aging.101143

**Published:** 2016-12-20

**Authors:** Kayla Daniell, Carmelo Nucera

**Affiliations:** ^1^ Laboratory of human thyroid cancers preclinical and translational research, Division of Experimental Pathology, Cancer Research Institute (CRI), Cancer Center, Department of Pathology, Beth Israel Deaconess Medical Center, Harvard Medical School, Boston, MA 02215 USA; ^2^ Department of Pathology, Center for Vascular Biology Research (CVBR), Beth Israel Deaconess Medical Center, Harvard Medical School, Boston, MA 02215 USA

**Keywords:** iodine, thyroid cancer, BRAFV600E, pre-clinical model, angiogenesis, tumor growth, VEGF, HIF-1, TGFB

## Abstract

Iodide is a micronutrient essential for thyroid hormone production. The uptake and metabolism of iodide by thyrocytes is crucial to proper thyroid function. Iodide ions are drawn into the thyroid follicular cell via the sodium-iodide symporter (NIS) in the cell membrane and become integrated into tyrosyl residues to ultimately form thyroid hormones. We sought to learn how an abnormal concentration of iodide within thyrocyte can have significant effects on the thyroid, specifically the surrounding vascular network. Insufficient levels of iodide can lead to increased expression or activity of several pathways, including vascular endothelial growth factor (VEGF). The VEGF protein fuel vessel growth (angiogenesis) and therefore enhances the nutrients available to surrounding cells. Alternatively, normal/surplus iodide levels can have inhibitory effects on angiogenesis. Varying levels of iodide in the thyroid can influence thyroid carcinoma cell proliferation and angiogenesis via regulation of the hypoxia inducible factor-1 (HIF-1) and VEGF-dependent pathway. We have reviewed a number of studies to investigate how the effect of iodide on angiogenic and oxidative stress regulation can affect the viability of thyroid carcinoma cells. The various studies outlined give key insights to the role of iodide in thyroid follicles function and vascular growth, generally highlighting that insufficient levels of iodide stimulate pathways resulting in vascular growth, and *viceversa* normal/surplus iodide levels inhibit such pathways. Intriguingly, TSH and iodine levels differentially regulate the expression levels of angiogenic factors. All cells, including carcinoma cells, increase uptake of blood nutrients, meaning the vascular profile is influential to tumor growth and progression. Importantly, variation in the iodine concentrations also influence BRAF^V600E^-mediated oncogenic activity and might deregulate tumor proliferation. Although the mechanisms are not well eluted, iodine concentrations and metabolism might have a crucial influence on thyroid carcinoma cell viability via regulation of different molecular pathways, including angiogenesis regulatory autocrine and microenvironment-mediated signals.

## INTRODUCTION

The synthesis of thyroid hormones is a fundamental process for cell metabolism and embryo-fetal development (e.g. brain). The proper production of thyroid hormones is dependent on many factors, including the metabolism of iodide within the thyroid gland [1]. Iodide is lacking in some environments but can be attained by consuming foods rich in this micronutrient [2]. The gastrointestinal track is capable of absorbing nearly 100% of iodide from consumed sources [3]. Upon absorption, iodide molecules enter the bloodstream and are delivered to various anatomical locations [4 5]. Upon sufficient iodide intake by a healthy adult, 70–80% of this natural micronutrient is localized in the thyroid [5].

### Role of iodine in normal thyroid cells

**Figure 1:**
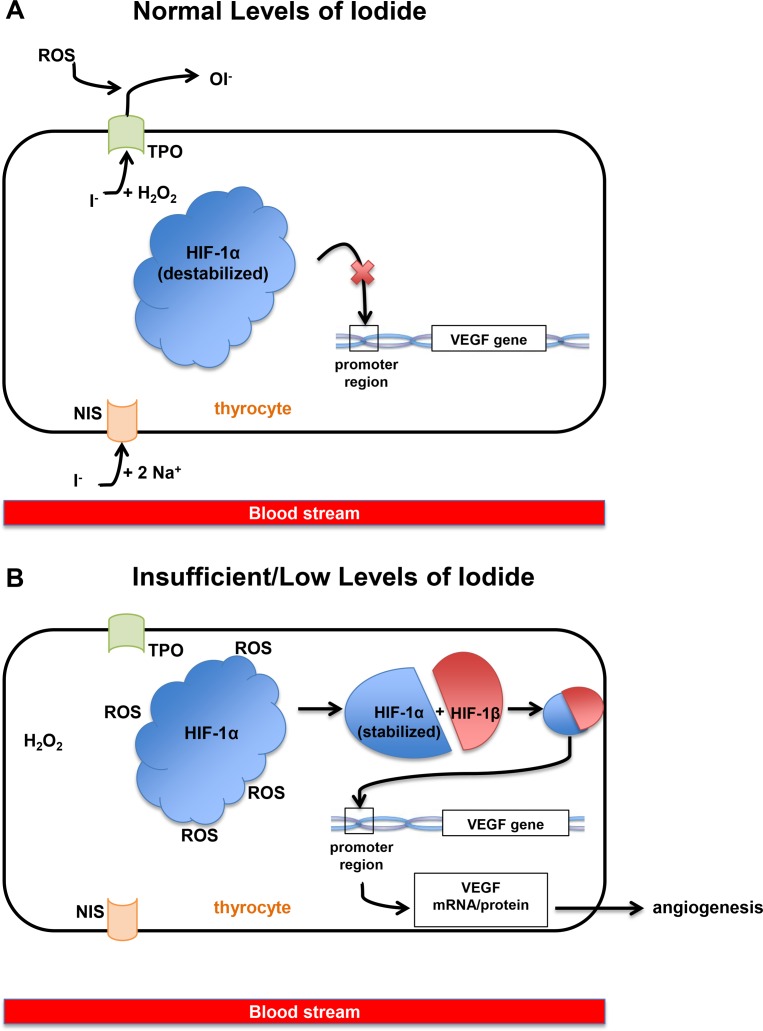
(**A**) When iodide exists at normal or sufficient levels in the blood, one iodide ion enters a thyrocyte along with sodium via NIS (sodium-iodide symporter) at the basolateral membrane of the cell. The iodide moves to the apical membrane of thyrocytes along with molecules of hydrogen peroxide. Finally, iodide is oxidized and HIF-1α (hypoxia inducible factor-1α) remains unstable and therefore unable to bind to the VEGF (Vascular Endothelial Growth Factor) promoter. Ultimately, this mechanism reduces the VEGF expression. (**B**) When iodide exists at insufficient levels in the blood, thyrocytes and thyroid follicles also contain low levels of iodide or no iodide, and cytoplasmic ROS (reactive oxygen species) levels remain high because there is no iodide to be oxidized. These ROS stabilizes HIF-1α, allowing the binding of HIF-1α with HIF-1β, which binds to the VEGF promoter and allows transcription of the VEGF gene.

Iodide is incorporated into thyrocytes via the sodium-iodide symporter (NIS) located in the basolateral membrane of thyrocytes (Fig. 1A). The iodide ion migrates to the apical membrane of the thyrocyte and is released into the follicular lumen to be oxidized by the membrane-bound thyroid peroxidase (TPO) enzyme (Fig. 1A). Sequentially, iodine is integrated into the tyrosyl residues of thyroglobulin (Tg), the protein upon which thyroid hormones are built, by TPO. Iodination of a single selected tyrosyl residue results in mono-iodotyrosine (MIT), and the iodination of two residues results in diiodotyrosine (DIT).

Triiodothyronine (T3) is formed by one molecule of MIT and one molecule of DIT, and thyroxine (T4) is formed by two molecules of DIT. These Tg structures are engulfed by thyrocytes and digested by lysozymes, resulting in T3 and T4 thyroid hormones that are secreted into the blood stream [4]. These thyroid hormones are crucial for facilitating numerous physiological processes throughout the body and are crucial for healthy development, specifically brain development [5].

### Effect of insufficient iodine on normal thyroid cells

Iodine deficiency in the thyroid is detrimental to thyrocytes due to the significance of iodine in the production of thyroid hormones. Insufficient uptake of iodide or deiodination of iodotyrosine residues via thyroidal iodotyrosine dehalogenase may be related to hypothyroidism and goiter [4]. Thyrocytes can overcome iodide deficiency by employing certain mechanisms to increase iodine levels. These mechanisms include trapping iodide in the NIS for storage, preferentially generating T3 over T4, or converting peripheral T4 into T3 [6]. Uncorrected irregularities in iodide concentration within thyrocytes can lead to changes in the thyroid vasculature with formation of abnormal vessels.

### Vascularity of the thyroid gland

The vascular networks of endocrine glands are extremely well suited for secretory functions. In the thyroid gland specifically, each individual follicle is encapsulated in its own capillary bed, and varying iodine content within the follicles can affect the surrounding vasculature of the gland [7]. Thyroid stimulating hormone (TSH), which contributes to thyroid hormone synthesis, can contribute to vessel growth (angiogenesis), however, TSH is not the only contributing factor [5]. The regulation of angiogenesis is rather complex and is likely dependent on the overall physiological status of the thyroid, specifically, on the production of thyroid hormones [8]. Mice with hypothyroidism showed an evident increase in capillary growth upon treatment with thyroid hormone, indicating that thyroid functionality has a positive influence on angiogenic activity [8].

Angiogenic growth is reliant on the expression of the vascular endothelial growth factor (VEGF) [9 10]. The VEGF family is a variety of genes involved in the stimulation of angiogenesis in and around the thyroid gland and other endocrine glands. Transcription of the VEGF gene relies on the hypoxia-inducible factor 1α/1β heterodimer as a promoter [5]. TSH can increase VEGF-A expression in thyrocytes [9] and therefore stimulate endothelial cell proliferation. Increasing vascularity surrounding thyroid follicles may also promote cell proliferation due to the increase in blood flow and micronutrient delivery.

### Effect of insufficient iodine on thyroid carcinoma angiogenesis

Iodine deficiency can induce angiogenesis independently from TSH by increasing the uptake of iodine and up-regulating the VEGF pathway [6]. Insufficient levels of iodide in thyrocytes increase the free reactive oxygen species (ROS) [5] (Fig.1B). These oxygen molecules stabilize hypoxia-inducible factor-1α (HIF-1α), allowing it to join with HIF-1β to form the heterodimer that binds to the promoter of the VEGF gene. Therefore, lack of iodide leads to increased transcription of the VEGF gene (Fig.1B). The resulting VEGF protein is subsequently secreted, expanding the surrounding vessels [5]. When mice on a low-iodine diet were administered a goitrogen treatment to block iodine uptake by thyrocytes, pro-angiogenic factors, such as VEGF-A and FGF2, showed increased levels of expression [7]. After 24 hours of treatment, control thyroids showed a drastic increase in VEGF-A and VEGF receptors. Both TPO and TSH levels remained normal for 6 days and increased at days 12 and 24. Tg levels were normal for the first 6 days and were depleted by day 12. This study concluded that vascular activation in early stages of induced hypothyroidism does not depend on TSH levels and are instead onset by the thyrocytes, which up-regulated expression of pro-angiogenic factors immediately upon sensing a decrease in iodine levels [7].

### Effect of excess iodine on thyroid angiogenesis

On the contrary, iodine in excess can have inhibitory effects on the thyroid gland, specifically on NIS expression and VEGF genes in FRTL-5 thyroid cells [11]. This study demonstrated that the mechanism of iodide suppression on NIS gene expression is transcriptional. These results suggest that excess iodide may affect thyroid vascular function via VEGF. Furthermore, when human thyroid follicles isolated from thyroids of patients with Graves’ disease were exposed to excess iodide, expression and secretion of VEGF-A and VEGF-B decreased and hormone synthesis declined, suggesting that this mechanism not only influences thyroid vascularity but also vascular permeability under hyperthyroid conditions [9]. Additionally, the excess dose of iodine increased activity and expression of anti-angiogenic factors such as angiostatin. Although tumor growth relies on abnormal angiogenesis which cooperates to intravascular invasion and metastasis, variation in secreted pro-angiogenic and anti-angiogenic factors levels may be related to the iodide concentrations and this subsequently can affect thyroid blood flow.

### Correlation between regulation of the VEGF pathway and thyroid varcinoma viability

The effect of iodine on the VEGF pathway and vascular formation provides insight to the viability of thyroid carcinoma cells with regards to angiogenesis. Gerard et al. suggested that iodine deficiency in the thyroid stimulates angiogenesis via over-expression of the VEGF pathway [6]. This finding consequently suggests that thyroid carcinoma cells benefit from iodine deficiency because the formation of new blood vessels allows for great nutrient uptake by the cancer and therefore their prosperity by altering the metabolic mechanisms of the VEGF pathway. Additionally, upon to exposure with excess iodide, both poorly differentiated or undifferentiated thyroid carcinoma cells, and rat thyroid follicular cells that conditionally express BRAF^V600E^ (PCCl3 line) showed a decrease in BRAF^V600E^-induced up-regulation of miR-17-92, including miR-19, blocking NOTCH signaling which confers proliferative advantage [12]. Overall, this study shows that high iodine exerts a protective influence over BRAF^V600E^-activated thyroid cells. Iodine might reduce acute BRAF^V600E^ oncogene induction and activity.

Collectively, all the above studies suggest that abnormal concentrations of iodide in thyrocytes clearly have a direct effect on the function of the thyroid as well as the surrounding vasculature and overall thyroidal blood supply. Due to the reliance of carcinoma cells on the nutrients found in the blood, altering the vasculature round thyroid follicles also affects thyroid carcinoma cell viability. Stimulation of VEGF expression and angiogenesis via lack of iodide has shown to be positively correlated to thyroid carcinoma cell viability [6]. Alternatively, excessive amounts of iodide in the thyroid may decrease VEGF expression in thyrocytes and increase anti-angiogenic factors, therefore reducing the blood supply to both the thyroid and depriving any surround carcinoma cells of important nutrients that it would normally retrieve from the blood.

### Concluding remarks

Although many relationships between molecular components of the thyroid (such as iodine and thyroid hormones) and angiogenesis are known, the mechanisms by which regulation is altered are not well-elucidated. Nonetheless, it is clear that intra-thyroidal iodine levels have a wide-spread impact on thyroid function, thyroidal blood supply, and also on thyroid carcinoma cell viability. We have reviewed in this article biological processes that are affected by abnormal iodide concentrations and the consequences on the thyroid and the surrounding thyroidal vasculature. Some mechanisms which might alter normal thyroid function include angiogenesis stimulation or suppression. Remarkably, angiogenesis is also known to contribute to thyroid cancer progression and is affected by either low or excess iodine. Also, iodine might exert effects against carcinoma cells, attenuating BRAF^V600E^ oncogene-mediated deregulation of some microRNAs. In summary, by understanding these mechanisms, we could explore new therapeutic options that target pathways metabolically influenced by iodine concentrations.
